# Patient‐reported outcome after treatment for definite Lyme neuroborreliosis

**DOI:** 10.1002/brb3.1595

**Published:** 2020-03-09

**Authors:** Randi Eikeland, Unn Ljøstad, Geir Helgeland, Geir Sand, Heidi Øyen Flemmen, Margrete Halvorsen Bø, Ludmila Nordaa, Jone Furulund Owe, Åse Mygland, Åslaug Rudjord Lorentzen

**Affiliations:** ^1^ Department of Pediatrics Sørlandet Hospital Trust Arendal Norway; ^2^ The Norwegian National Advisory Unit on Tick‐borne Diseases Arendal Norway; ^3^ Department of Neurology Sørlandet Hospital Trust Kristiansand Norway; ^4^ Department of Clinical Medicine University of Bergen Bergen Norway; ^5^ Department of Neurology Møre and Romsdal Hospital Trust Molde Norway; ^6^ Department of Infectious Diseases Stavanger University Hospital Stavanger Norway; ^7^ Department of Neurology Telemark Hospital Trust Skien Norway; ^8^ Department of Neurology Oslo University Hospital Oslo Norway; ^9^ Department of Neurology Helse Fonna Trust Haugesund Norway; ^10^ Department of Neurology Haukeland University Hospital Bergen Norway; ^11^ Department of Rehabilitation Sørlandet Hospital Trust Kristiansand Norway; ^12^ Present address: Eide legesenter as Eide Norway; ^13^ Present address: N.K.S. Jæren distriktspsykiatriske senter AS Bryne Norway

**Keywords:** fatigue, neuroborreliosis, patient‐reported outcome measures

## Abstract

**Objective:**

To chart patient‐reported outcome measures (PROMs) in Norwegian patients treated for definite neuroborreliosis (NB).

**Material and Methods:**

Adult patients treated for definite NB 1–10 years earlier supplied demographics, symptoms and treatment during NB, and answered validated questionnaires; Fatigue Severity Scale (FSS), Hospital Anxiety and Depression Scale (HADS), health‐related quality of life questionnaire (RAND‐36), and Patient Health Questionnaire (PHQ‐15).

**Results:**

A higher proportion of NB‐treated persons reported severe fatigue, defined as FSS score ≥ 5, than in Norwegian normative data, but when removing persons with confounding fatigue associated comorbidities (*n* = 69) from the analyses, there was no difference between groups. Physical health‐related quality of life (RAND‐36 PCS), mean FSS score, proportions of persons reporting moderate or severe somatic symptom burden (PHQ‐15 score ≥ 10), anxiety (HADS‐A ≥ 8), or depression (HADS‐D ≥ 8) did not differ between NB‐treated persons and reference scores. Mental health‐related quality of life (RAND‐36 MCS) was poorer than in normative data (47.1 vs. 53.3), but associated with anxiety, depression and current moderate or severe somatic symptom burden, and not with NB characteristics.

**Conclusions:**

Results on validated PROM questionnaires measuring fatigue, anxiety, depression, self‐reported somatic symptom burden, and physical health‐related quality did not differ between persons treated for definite NB 1–10 years earlier and reference scores. NB‐treated persons tended to report a slightly poorer mental health‐related quality of life than found in normative data, but when adjusting for confounders the causative connection is questionable. Overall, the long‐term prognosis of definite NB seems to be good.

## Graphical abstract

This study includes a big cohort of patients treated for definite neuroborreliosis according to current diagnostic guidelines, and the patients long term reported outcomes are presented.

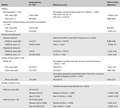

## 1 INTRODUCTION

Most patients recover well after treated neuroborreliosis (NB; Obel et al., 2018), but some report residual symptoms as pain, paresthesias, fatigue, and cognitive deficits (Eikeland, Ljostad, Mygland, Herlofson, & Lohaugen, 2012; Eikeland, Mygland, Herlofson, & Ljostad, 2011; Knudtzen, Andersen, Jensen, & Skarphedinsson, 2017; Ljøstad & Mygland, 2010). Almost all aspects of the phenomenon postborreliosis symptoms are debated, including its existence, prevalence, cause, spectrum, and impact (Halperin, 2017). In a meta‐analysis of 34 studies, Dersch, Sommer, Rauer, and Meerpohl (2016) found that about 28% of patients treated for NB had residual symptoms, most often pain. The prevalence was highest in studies using diffuse NB case definition. The authors debate whether the results are biased and if reports of debilitating fatigue and cognitive impairment after NB could “be an artifact of unspecific case definitions in single studies”. It is also of note that several of the included studies did not use validated and standardized outcome measures. For future studies, it was recommended to apply strict case definitions and to put more emphasis on impact of residual symptoms.

In the current study, we aimed to chart long‐term prognosis of NB by collecting patient‐reported outcome measures (PROMs) using validated questionnaires in persons treated for definite NB according to current diagnostic guidelines (Mygland et al., 2010).

## 2 MATERIAL AND METHODS

### 2.1 Study design

Cross‐sectional study.

### 2.2 Patient recruitment

All departments of Neurology in Norway were invited to identify and recruit patients by reviewing medical records. Inclusion criteria were adult patients (≥18 years) treated for definite NB according to the EFNS diagnostic guidelines (neurological symptoms suggestive of NB without other obvious reasons, pleocytosis in CSF and intrathecal *Borrelia burgdorferi* antibody production; Mygland et al., 2010) between 2006 and 2014. Twelve out of 18 invited hospitals agreed to participate. A contact person at each department reviewed the medical records for eligible patients (*n* = 598) and invited them per letter to fill in the PROM questionnaires, preferred web‐based, but optional paper‐based during 2015 to 2016.

### 2.3 Variables and questionnaires

Year of NB diagnosis and CSF cell count were obtained from medical records.

### 2.4 Patient‐reported information about education level

Questions about education were obtained by reuse of formulations from the Nord‐Trøndelag Health Study 3 (HUNT3), which is a Norwegian community‐based survey (Krokstad et al., 2013).

### 2.5 Patient‐reported information about NB

#### 2.5.1 Symptoms

The patients were asked if they had experienced any of the following symptoms during their NB; Headache, fatigue, malaise, general pain, radiating pain from neck or back, facial palsy, paresis, numbness, memory or concentration problems, dizziness or unsteadiness, diplopia, or other. A sum score of symptom burden during NB was provided by giving each “yes” one point (max score 12).

#### 2.5.2 Treatment

The patients were asked if they were treated with orally or intravenously administered antibiotics or both, and for how many weeks (by ticking off 1–6). For both questions, “I do not remember” was an optional answer.

### 2.6 Patient‐reported information about comorbidity

The patients were asked if they had ongoing or previous any of the following comorbidities: diabetes mellitus, metabolic disease, rheumatoid arthritis, fibromyalgia, systemic disease (sarcoidosis, systemic lupus erythematosus, or Sjögren's syndrome), other musculoskeletal disease, multiple sclerosis, Parkinson's disease, dementia, epilepsy, stroke, polyneuropathy, other neurological diseases, cancer, heart disease, asthma or chronic obstructive lung disease, allergy, chronic fatigue syndrome, depression or anxiety, other psychiatric disease, celiac disease, osteoporosis, kidney disease, other diseases not specified further. If they answered yes to one or more, the patients were classified as suffering from comorbidity. A sum score was provided by giving each “yes” one point (max score 24).

### 2.7 Patient‐reported outcome measures (PROMs)

#### 2.7.1 Fatigue

*Fatigue Severity Scale (FSS)* is a validated questionnaire (Krupp, LaRocca, Muir‐Nash, & Steinberg, 1989) that measures level of agreement (1–7) with nine statements about fatigue. The final score represents the mean value of the nine items. Scores ≥ 5 are regarded as severe fatigue (Lerdal, Wahl, Rustøen, Hanestad, & Moum, 2005).

#### 2.7.2 Somatic symptom burden during the last 4 weeks

*The Patient Health Questionnaire ‐15 (PHQ‐15)* is a validated questionnaire that charts prevalence and intensity of 15 somatic symptoms (stomach pain, back pain, pain in arms/legs/joints, menstrual cramps, or other problems with periods (women only), headache, chest pain, fainting spells, feeling of heartpounds or race, shortness of breath, pain or problems during sexual intercourse, constipation/loose bowels/diarrhea, nausea/gas/indigestion, feeling tired/having low energy, trouble sleeping) during the last 4 weeks (Kroenke, Spitzer, & Williams, 2002). The answers are graded “not bothered at all” (0 points), “bothered a little” (1 point), and “bothered a lot” (2 points). Sum score ranges from 0 to 28 for men and from 0 to 30 for women.

Scores 5–9 points, 10–14 points, and 15–30 points indicate mild, moderate, and severe somatic symptom burden, respectively (Zijlema et al., 2013). We translated the PHQ‐15 questionnaire into Norwegian based on the Swedish version (Nordin, Palmquist, & Nordin, 2013). A missing value for a PHQ‐15 item was replaced with the average value of the other items if the number of missing values did not exceed three items (*n* = 3; Kroenke et al., 2002).

#### 2.7.3 Anxiety and depression

*Hospital Anxiety and Depression Scale (HADS)* is a validated questionnaire measuring symptoms of anxiety and depression (Bjelland, Dahl, Haug, & Neckelmann, 2002). It includes seven anxiety items and seven depression items scored on a four‐point Likert‐like scale. Scores ≥ 8 on the subscales indicate a need of further assessment of possible anxiety and/or depression (Leiknes, Dalsbo, & Siqveland, 2016). A missing value in either the anxiety or depression items was replaced with the average value of the other items if the number of missing values did not exceed one item in each subscore (*n* = 2).

#### 2.7.4 Health‐related quality of life

*RAND‐36* is a validated questionnaire that measures health‐related quality of life (Ware, 2000). It consists of 36 questions comprising eight multi‐item scales: mental health, vitality, bodily pain, general health, social function, physical function, and physical and emotional role. These domains are then combined into physical and mental component summary scales (PCS and MCS).

### 2.8 Statistical analysis

Results are given as proportions, mean with standard deviations (*SD*), or median with range as appropriate. To calculate 95% confidence intervals of differences, we used online calculators http://vassarstats.net/prop2_ind.html (proportions) and https://www.socscistatistics.com/confidenceinterval/Default4.aspx (means). Selected variables, considered to be of importance, were entered into a multivariate linear regression analysis with mental health‐related quality of life (MCS) score as the dependent variable. *p*‐Values < .05 were considered statistically significant. All statistical analyzes were performed using SPSS version 25 (SPSS Inc.).

### 2.9 Ethics

The study was approved by the Norwegian Regional Committees for Medical and Health Research Ethics, and all patients gave a written informed consent (REK 2013/1325).

## 3 RESULTS

Out of 598 invited NB patients, 307 gave a written informed consent to participate in the study, and 258 returned a satisfactorily completed questionnaire (228 web‐based, 30 paper), yielding a response rate of 43%. Of ethical reasons (lack of informed consent), we could not obtain data from the nonresponders to detect meaningful differences between those who responded and those who did not.

Patient characteristics at the time of filling the questionnaire are shown in Table 1.

**Table 1:** Demographic features of the study population at the time of filling out the questionnaire

	Study patients *n* = 258
Age, mean (*SD*)	62.4 (12.7)
Females, *n* (%)	129/258 (50)
Years since treatment for NB, mean (range)	5.0 (1–10)
High educational level (university or college), *n* (%)	111/257 (43)

Abbreviation: *SD*, standard deviation.

A total of 185 NB‐treated patients (71%) reported to have at least one of the listed comorbidities, of which the most frequently reported were other musculoskeletal diseases (nonrheumatic, not fibromyalgia; 17%), allergy (14%), depression or anxiety (11%), and other not defined diseases (20%). Sixty‐nine persons (27%) reported diseases often associated with fatigue as multiple sclerosis (3), systemic disease sarcoidosis, systemic lupus erythematosus, or Sjögren's syndrome (4), rheumatoid arthritis (9), fibromyalgia (10), chronic fatigue syndrome (4), Parkinson's disease (1), thyroid dysfunctions (22), or cancer (28). Mean number of reported comorbidities was 1.4 (*SD* = 1.3).

Patient‐reported information on clinical characteristics and treatment of the NB are shown in Table 2. Median CSF leukocyte count at the time of diagnosis was 171 10^6^/L (range 5–3498 10^6^/L), and 75% of the CSF samples had a leukocyte count between 62 and 296 10^6^/L.

**Table 2:** Patient‐reported information about their Neuroborreliosis; clinical characteristics and treatment

	Study patients, *n* = 258
Reported symptoms, *n* (%)	
Radiating pain	120 (47)
General pain	117 (45)
Fatigue	116 (45)
Facial palsy	109 (42)
Malaise	108 (42)
Dizziness and/or unsteadiness	91 (35)
Headache	89 (35)
Numbness in arm and/or leg	80 (31)
Memory and/or concentration problems	75 (29)
Paresis in arm and/or leg	48 (19)
Diplopia	31 (12)
Other	7 (3)
Total symptom burden (max score 12), median (range)	4 (1–11)
Administration route of antibiotics, *n* (%)	
Exclusively oral	76 (30)
IV or combined IV and oral	181 (70)
Unknown	1
Treatment duration > 2 weeks, *n* (%)	153/244 (63)

Patient‐reported outcome measures, relevant reference scores, and estimated 95% CI of the differences between our findings and reference scores are shown in Table 3. A higher proportion of NB‐treated persons reported severe fatigue, defined as FSS score ≥ 5, than in Norwegian normative data, but when removing persons with confounding fatigue associated comorbidities (*n* = 69) from the analyses, there was no difference between groups. Physical health‐related quality of life (RAND‐36 PCS), mean FSS score, proportions of persons reporting moderate or severe somatic symptom burden (PHQ‐15 score ≥ 10), anxiety (HADS‐A ≥ 8), or depression (HADS‐D ≥ 8) did not differ between NB‐treated persons and reference scores. Mental health‐related quality of life (RAND‐36 MCS) was poorer among NB‐treated patients than in normative data. To further analyze this finding, we did a linear regression and found that poorer mental health‐related quality of life was associated with HADS‐A ≥ 8 (*p* < .001), HADS‐D ≥ 8 (*p* < .001), and PHQ‐15 ≥ 10 (*p* = .024), but not with sex, age, years since NB diagnosis, education level, treatment administration route, treatment duration, CSF cell count at diagnosis, burden of symptoms at diagnosis, memory and/or concentration problems at diagnosis, comorbidity, or FSS ≥ 5.

**Table 3:** Patient‐reported outcome measures (PROMs) as compared to reference scores (normative data or other relevant reference groups)

PROMs	Study patients *N* = 258	Reference scores	95% CI of the difference
Fatigue			
All responders *n* = 253		Norwegian normative data (Lerdal et al., 2005) *n* = 1,893 FSS, mean: 4.0, *SD* 1.3 FSS score ≥ 5:23.1%	
FSS, mean (*SD*)	3.84 (1.88)		−0.002 to 0.34
FSS score ≥ 5	80 (32%)		**0.03 to 0.15**
Responders without fatigue associated comorbidities *n* = 184			
FSS, mean (*SD*)	3.63 (1.84)		**0.2 to 0.6**
FSS score ≥ 5	50 (27%)		−0.02 to 0.11
Anxiety and depression			
Anxiety subscore		Data from HUNT 2 and HUNT 3 (Leiknes et al., 2016)	
HADS‐A, mean (*SD*)	4.6 (3.7)	4.0 (3.3) *n* = 39,277	**0.20 to 1.10**
HADS‐A score ≥ 8	50/257 (19%)	15% *n* = 7,014	−0.002 to 0.1
Depression subscore			
HADS‐D, mean (*SD*)	3 (3.0)	3.3 (2.9) *n* = 39,573	−0.26 to 0.46
HADS‐D score ≥ 8	26/255 (10%)	11.6% *n* = 7,014	−0.03 to 0.05
Health‐related quality of life			
RAND‐36		Norwegian normative data (50–59 years; Garratt & Stavem, 2017) *n* = 947	
PCS, mean (*SD*)	46.5 (9.7)	47.8 (10.7)	−0.16 to 2.76
MCS, mean (*SD*)	47.1 (9.9)	53.3 (8.7)	**4.95 to 7.45**
		Norwegian population (unpublished data; Thortveit, Lorentzen, Ljøstad, & Mygland, 2019) *n* = 2,872	
MCS, mean (*SD*)	47.1 (9.9)	48.5 (10.7)	**0.03 to 2.77**
Somatic symptom load			
		Swedish normative data (Nordin et al., 2013)	
PHQ‐15, mean (*SD*)	All 6.2 (4.7)		
	Women 6.8 (4.9)	Women 7.2 (4.8) *n* = 1,898	−0.702 to 1.102
	Men 5.7 (4.3)	Men 5.3 (4.4) *n* = 1,508	−0.199 to 1.399
PHQ‐15 score ≥ 10	All 57/245 (23%)		
	Women 35/117 (30%)	Women 26.4% *n* = 1,898	−0.03 to 0.15
	Men 22/128 (17%)	Men 14.8% *n* = 1,508	−0.005 to 0.15

Abbreviations: FSS, Fatigue Severity Scale; HADS, Hospital Anxiety and Depression Scale; MCS, Mental component summary scales; PCS, Physical component summary scales; PHQ‐15, Patient Health Questionnaire‐15; RAND‐36, Short Form Health Survey.

Bold indicate statistically significant values.

## 4 DISCUSSION

The main results in the present study were that in our group of 258 Norwegian patients treated for definite NB one to ten years earlier the prevalence of severe fatigue, substantial symptomatic symptom burden, anxiety, and depression did not differ from relevant reference groups, and reported physical health‐related quality of life was similar to normative data. Mental health‐related quality of life tended to be slightly poorer among NB‐treated patients, but was associated with anxiety, depression and presence of moderate or severe current subjective somatic symptom burden and not with any NB‐related factors. Based on our findings, we therefore state that long‐term prognosis of definite NB seems to be good.

Residual symptoms after treated NB is a much debated phenomenon (Halperin, 2017). Some have found residual symptoms in up to 50% (Eikeland et al., 2012; Eikeland et al., 2011; Knudtzen et al., 2017; Ljøstad & Mygland, 2010), while others have found that NB has no substantial effect on long‐term survival, health, or educational and social functioning (Obel et al., 2018). Some of the disagreement may be explained by different study designs and poor case definitions (Dersch et al., 2016).

Our study design does not permit any final conclusions or detection of causal connections, but our results give a substantial contribution to the debate. It is interesting that when we apply strict case definition and validated PROMs there seem to be very few residual symptoms 1–10 years after adequate treatment for NB.

Some would argue that a recruitment rate of 43% might have caused a selection bias, and that the time from acute disease might have caused a recall bias, and that our findings therefore are unreliable. In our opinion, this is not a big error source, as one would expect both these potential biases to lead to over reporting of complaints rather than under reporting.

Further, comparison with normative data should be done with caution and have several caveats. Among other things, the populations may differ regarding gender, age, education level, and comorbidity, and also regarding cultural conditions, setting, and patient expectations. Nevertheless, the comparisons can express trends. Another study (Eikeland et al., 2011) using validated questionnaires have included matched control persons for comparison. They found poorer physical and mental health‐related quality of life, and more fatigue among NB‐treated persons than among controls, but case definitions in this study were less strict, as 16/50 (32%) were classified as possible NB.

The mean age (62 years) in our cohort was rather high, and a substantial proportion reported to have comorbidity (71%), thus one would expect a certain amount of reported symptoms due to other reasons than previous NB. Unfortunately, we did not ask whether the patients attributed their current complaints to the previous NB or to other comorbidities. Instead, we adjusted for this confounder by removing persons with fatigue associated comorbidity when comparing fatigue scores. By doing this, there was no difference between NB‐treated patients and normative data, and mean FSS score changed from similar to lower in the NB‐treated group. We therefore think that even if the question about causality between the PROMs and NB is impossible to answer with our study design, it is proper to state that NB‐treated persons do not suffer from more fatigue than found in normative data.

Previous prospective studies have found that residual symptoms after treated NB is associated with factors as pretreatment symptom duration > 6 weeks, pretreatment burden of symptoms and findings, pretreatment CSF leukocyte count, and female gender (Eikeland, Mygland, Herlofson, & Ljøstad, 2013; Knudtzen et al., 2017; Ljøstad & Mygland, 2010). We did not find any association between the only PROM that differed between NB‐treated persons and reference data, namely mental health‐related quality of life, and pretreatment CSF leukocyte count and symptom burden. Instead, poor mental health‐related quality of life was associated with anxiety, depression, and moderate or severe symptom burden last 4 weeks, and we therefore assume that the impairment was caused by other factors than NB.

## 5 CONCLUSION

Results on validated PROM questionnaires measuring fatigue, anxiety, depression, self‐reported somatic symptom burden, and physical health‐related quality did not differ between persons treated for definite NB 1–10 years earlier and reference scores. NB‐treated persons tended to report a slightly poorer mental health‐related quality of life than found in normative data, but when adjusting for confounders the causative connection is questionable. Although our study has several shortcomings, we conclude that the long‐term prognosis of definite NB seems to be good.

## CONFLICT OF INTEREST

The authors have no conflict of interest.

## AUTHOR CONTRIBUTIONS

ÅL was responsible for conception and design of the study, for data acquisition, analysis and interpretation. RE, UL, and ÅM were responsible for conception and design of the study, data analysis and interpretation. GH, GS, HØF, MHB, LN, JFO included patients in the studies, and all authors revised and approved the final version of the manuscript.

## Data Availability

The data that support the findings of this study are available from the corresponding author upon reasonable request.
